# Diverse roles played by “*Pseudomonas fluorescens* complex” volatile compounds in their interaction with phytopathogenic microrganims, pests and plants

**DOI:** 10.1007/s11274-023-03873-0

**Published:** 2024-01-28

**Authors:** Aida Raio

**Affiliations:** National Research Council, Institute for Sustainable Plant Protection (CNR-IPSP), Via Madonna del Piano, 10., 50019 Sesto Fiorentino, FI Italy

**Keywords:** Biocontrol, Biopesticide, Fluorescent *Pseudomonas*, Induced systemic resistance, Microbial volatile compounds, Plant growth promotion

## Abstract

*Pseudomonas fluorescens* complex consists of environmental and some human opportunistic pathogenic bacteria. It includes mainly beneficial and few phytopathogenic species that are common inhabitants of soil and plant rhizosphere. Many members of the group are in fact known as effective biocontrol agents of plant pathogens and as plant growth promoters and for these attitudes they are of great interest for biotechnological applications. The antagonistic activity of fluorescent *Pseudomonas* is mainly related to the production of several antibiotic compounds, lytic enzymes, lipopeptides and siderophores. Several volatile organic compounds are also synthesized by fluorescent *Pseudomonas* including different kinds of molecules that are involved in antagonistic interactions with other organisms and in the induction of systemic responses in plants. This review will mainly focus on the volatile compounds emitted by some members of *P. fluorescens* complex so far identified, with the aim to highlight the role played by these molecules in the interaction of the bacteria with phytopathogenic micro and macro-organisms and plants.

## Introduction

### General features of microbial volatile compounds

Microorganisms are able to synthesize a large number of volatile molecules that are propagated in air and liquid media, as well as in soil, acting as infochemicals for inter- and intraspecific communication and showing their effect at short and long distances (Veselova et al. 2019). Volatile organic compounds are small (less than 300 Da), odorous, carbon-based molecules with low water solubility and high vapour pressure that make them available in a gaseous status in the normal ambient conditions (Schulz-Bohm et al. 2017; Tilocca et al. 2020). The totality of volatile compounds produced by an organism or an ecosystem is the volatilome (Bailly and Weisskopf 2017), a term that includes molecules originated from different biochemical pathways and showing a broad chemical complexity (Maffei et al. 2011). In fact, microbial volatile metabolites include both inorganic molecules such as CO, CO_2_, N_2_, NH_3_, H_2_S, HCN and a wide variety of organic compounds involved in many biological functions. During the last years microbial volatile organic compounds (mVOCs) of many bacterial species have been characterized, evidencing the variability of blend composition and the complexity of the role exerted by these molecules not only at intra- but even at inter-kingdom level (Schulz-Bohm et al. 2017). The composition of mVOC blend is affected by several factors: the emitting microbial species, the availability and type of nutrients, temperature, radiation, relationship with the other microorganisms and the type of ecosystem (Tilocca et al. 2020). Results of in vitro studies on bacterial volatilome are strongly related to the characteristics of the growth medium since the carbohydrate and protein availability induces relevant metabolic changes (Blom et al. 2011). Moreover, it has been evidenced that the amount and type of VOCs synthesized by *P. fluorescens* WR1 was different if the bacterium was grown on agar medium, sterilized or natural soil (Raza et al. 2016) evidencing that the soil microbial community represents an important factor affecting volatile synthesis.

### Chemical characteristics, biosynthesis and regulation of microbial volatile compounds

**Fig. 1:**
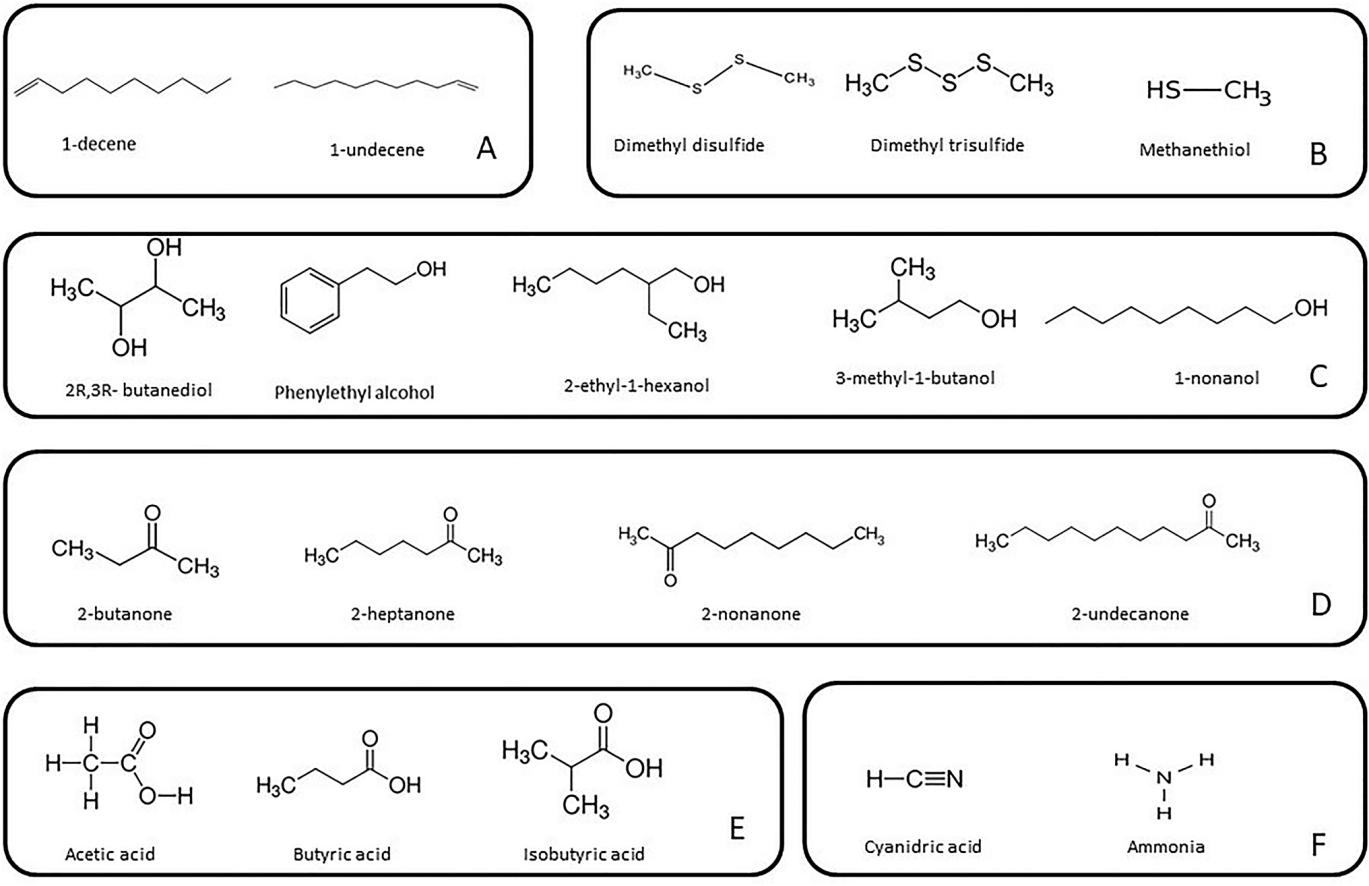
Chemical structure of the main volatile compounds emitted by strains belonging to *Pseudomonas* *fluorescens* complex. A: alkenes; B: sulfur compounds; C: alcohols; D: ketones; E: organic acids; F: inorganic compounds

**Fig. 2:**
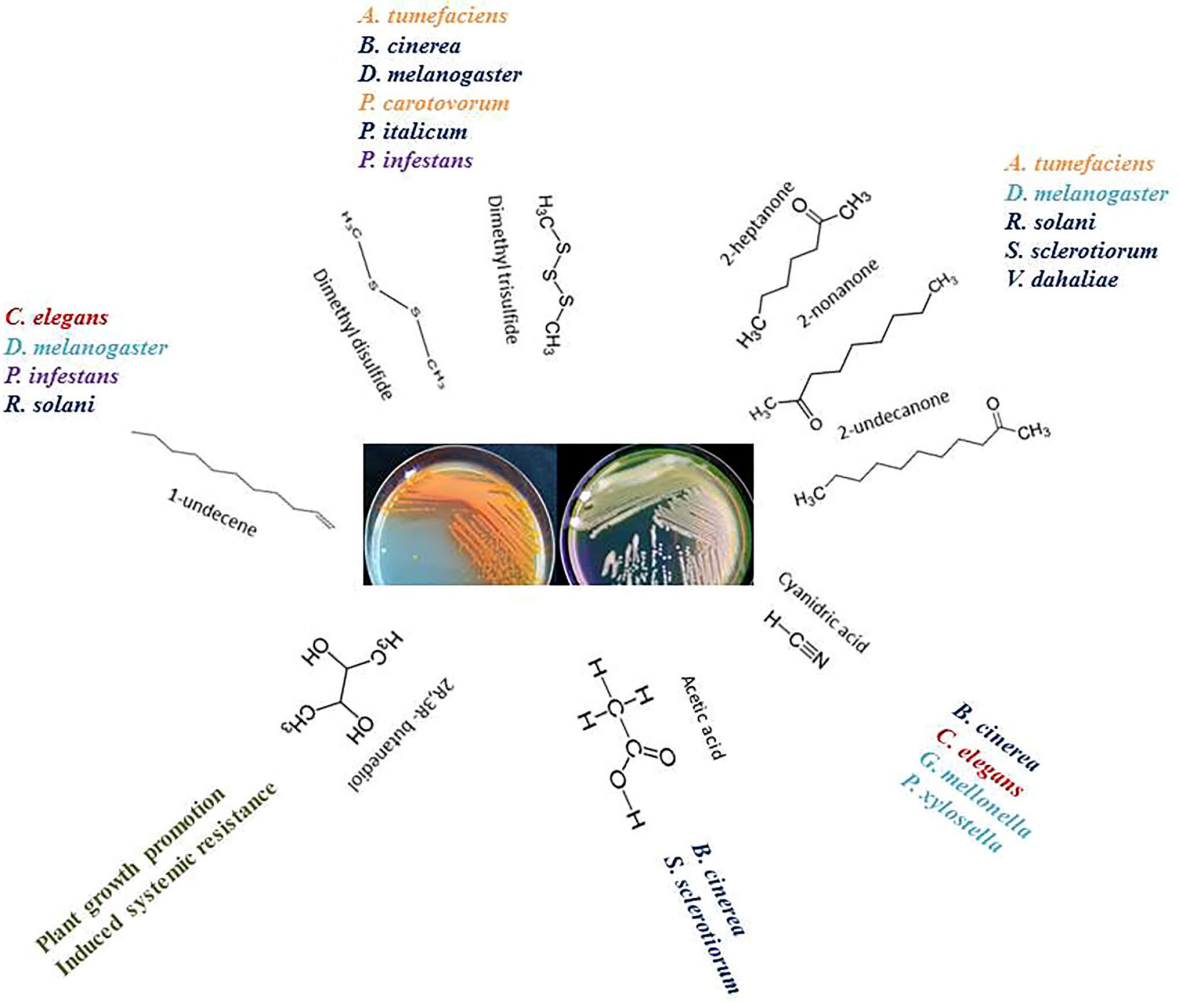
Activity of the main volatiles produced by strains belonging to *Pseudomonas* *fluorescens* complex on different organisms: blue: fungi; purple: oomycetes; orange: bacteria; red: nematodes; light blue: insects; green: plants

mVOCs belong to different chemical classes including alkanes, alkenes, alcohols, cyclohexanes, furanes, hydrocarbons, ketones, organosulfur compounds, terpenes, and thioesters. Many VOCs are produced by microorganisms during primary metabolism and energy generation. The underlying biosynthetic pathways are aerobic heterotrophic carbon metabolism, fermentation, amino acid degradation, terpenoid biosynthesis and sulphur reduction (Penuelas et al. 2014). Many microbial volatile blends contain C6 to C16 hydrocarbon compounds, mainly alkenes and aliphatic alcohols and ketones. These compounds are typically the result of fatty acid metabolism. In *Pseudomonas* genus several species are able to synthesize the alkene 1-undecene, that is related to modulation of *Arabidopsis thaliana* growth and pathogen/pest inhibition (Fig. 1; Table 1). The 1-undecene biosynthesis pathway was evidenced in *Pseudomonas aeruginosa* and it was shown that the alkene is obtained from the degradation of medium-length fatty acids (Rui et al. 2014). Sulfur volatile compounds like DMS, DMDS, DMTS and MT are methionine derivatives and are a group of volatiles very common among *Pseudomonas* mainly involved in the control of bacterial and fungal phytopathogens (Fig. 1; Table 1). Among the inorganic volatile compounds, HCN is synthesized from glycine by HCN synthase in several *Pseudomonas* species (Veselova et al. 2019) and it inhibits the growth of plant pathogens, insects and nematodes (Table 1; Fig. 2).

**Table 1:** Main volatile compounds produced by strains belonging to *Pseudomonas fluorescens* complex, target micro- and macro-organisms and effect on plants

	Volatile compounds	VOC emitter species	Target organisms	Effect on plants	References
*P. fluorescens group*	1-undecene	*P. fluorescens* R76	*Phytophthora infestans*	n.d.	Hunziker et al. (2015)
	Formamide and N,N-dimethyl- formamide	*P. fluorescens* ALEB7B	n.d.	*Atractylodes lancea* growth promotion	Zhou et al. (2016)
	1-undecene, DMDS; MT	*P. fluorescens* B4117, *P. fluorescens* Q8r1-96	*Agrobacterium* spp.	n.d.	Dandurishvili et al. (2011)
	1-undecene	*P. fluorescens* L13-6-12	*Rhizoctonia solani*	n.d.	Kai et al. (2007)
	Unidentified	*P. fluorescens* MGR 12	*Fusarium proliferatum*	n.d.	Cordero et al. (2014)
	2-Butanone, 13- Tetradecadien-1-ol; 2-Methyl- n-1-tridecene	*P. fluorescens* SS101	n.d.	*Nicotiana tabacum* growth promotion	Park et al. (2015)
	DMS, DMDS, DMTS, DMHDA	*P. fluorescens* UM16, UM240, UM256, UM270	*Botrytis cinerea*	*Medicago truncatula* growth promotion	Hernandez-Leon et al. (2015)
	Benzothiazole, 1-methyl naphtalene; m-xylene; benzaldeide	*P. fluorescens* WR1	*Ralstonia solanacearum*	n.d.	Raza et al. (2016)
	DMDS, DMTS. DMDS, DMTS, geranyl formate, acetic acid, butyric acid, 2-methylbutyric acid, isobutyric acid, isovaleric acid	*P. fluorescens* ZX	*Penicillium italicum* *Botrytis cinerea*	n.d.	Wang et al. (2020, 2021); Zhong et al. (2021)
	1-undecene, DMDS,1-decene, tridecane,	*P. simiae* PIC F7	*Verticillium* spp.	Systemic resistance in *A. thaliana*	Maldonado-Gonzalez et al. (2015); Montes-Osuna et al. (2022)
	n.d.	*P. simiae* AU	n.d.	Soybean growth promotion and tolerance to salt stress	Vaishnav et al. (2015)
	n.d.	*P. trivialis*	Several fungal species	*A. thaliana* growth inhibition	Vespermann et al. (2007)
	Undecadiene, Undecene, Benzyloxybenzonitrile	*P. trivialis* 3Re2-7	*Rhizoctonia solani*	n.d.	Kai et al. (2007)
	3- pentadecenenitrile; methyltridec-3-enenitrile	*P. veronii* R02	*Bacillus subtilis*, *Streptococcus aureus*, *Micrococcus luteus*. *Mucor hiemalis*	n.d.	Montes Vidal et al. (2017)
*P. corrugata* group	HCN	*P. brassicacearum* DF41	*Caenorhabditis elegans*	n.d.	Nandi et al. (2016)
	DMDS, DMTS	*P. brassicacearum* EnPb	*Pectobacterium carotovorum*	n.d.	Aghdam et al. (2023)
	Acetic acid, 2-nonanone, 2-undecanone	*P. brassicacearum* USB	*Sclerotinia sclerotiorum*	n.d.	Giorgio et al. (2015)
	HCN	*P. corrugata* CFBP5454; *P. mediterranea* CFBP5457	*Botrytis cinerea*	n.d.	Strano et al. (2017)
	Dodecane, tetradecane, hexadecane, o-xylene, benzene, 1,3 dimethyl	*P. kilonensis*	*Agrobacterium tumefaciens*	n.d.	Etminani et al. (2022)
*P. chlororaphis* group					
	2-undecanone and 1-nonanol. 2-undecanone	*P. aurantiaca* ST-TJ4	*Verticillium dahliae* *Phytophthora cinnamomi*	n.d.	Ni et al. (2022); Zhang et al. (2022)
	HCN, Methanethiol, DMDS	*P. chlororaphis* M71	*Seiridium cardinale, Clavibacter* *michiganensis*	n.d.	Raio et al. (2020)
	2R, 3R-butanediol	*P. chlororaphis* O6	n.d.	*Nicotiana tabacum* systemic resistance to *Erwinia carotovora*; *A. thaliana* systemic tolerance to drought	Han et al. (2006); Cho et al. (2008)
	Nonanal, benzothiazole and 2-ethyl-1-hexanol. HCN	*P. chlororaphis* PA23	*Sclerotinia sclerotiorum* *Caenorhabditis elegans*	n.d.	Fernando et al. (2005); Athukorala et al. (2010); Nandi et al. (2015)
	3-methyl-1-butanol, phenylethyl Alcohol, 2-methyl-1-butanol	*P.chlororaphis* SPS-41	*Ceratocystis fimbriata*	n.d.	Zhang et al. (2019)
	1-undecene, 2-nonanone, and 2- undecanone, 2 heptanone, DMDS	*P.chlororaphis* 449	*A. tumefaciens*, S*ynechococcus* sp., *Rhizoctonia solani*, *Caenorhabditis elegans*, *Drosophila melanogaster*	n.d.	Popova et al. (2014)
	1-undecene, 2-nonanone, and 2- undecanone, 2-heptanone, DMDS	*P.chlororaphis* 449	n.d.	*A. thaliana* growth inhibition	Plyuta et al. (2021)
	Isovaleric acid, phenylpropanedione, DMTS, MMTS, HCN	*P.chlororaphis* R47,	*Phytophthora infestans*	n.d.	De Vrieze et al. (2015); Anand et al. (2020)
	HCN	*P. chlororaphis* PCL1391	*Plutella xylostella; Galleria mellonella*	n.d.	Flury et al. (2017)
*P. protegens* group	DMTS; 2-ethylhexanol; Ammonia HCN	*P. protegens* CHA0 *P. protegens* CHA0	*Heterobasidion abietinum;* *Plutella xylostella; Galleria mellonella*	n.d. n.d.	Prigigallo et al. (2021); Flury et al. (2017)
	1,3-benzothiazole, 2-ethylhexanol, methyl thiocyanate, DMDS	*P. protegens* CLP-6	*Ralstonia solanacearum*	n.d.	Zhao et al. (2023)

*n.d.* not determined

In *Pseudomonas*, the production of various secondary metabolites and extracellular enzymes involved in plant pathogenicity, biocontrol, ecological fitness and tolerance to stress, is under the regulation of the GacS/GacA two-component regulatory system (Heeb and Has 2001; Lapouge et al. 2008). The involvement of the GacA/GacS regulatory system in the synthesis of VOCs was shown for the first time by Han et al. (2006). Indeed, it was evidenced the *gac*S-dependent production of 2R, 3R-butanediol in *P. chlororaphis* O6, a VOC responsible of the induction of systemic resistance against *Erwinia carotovora* in tobacco plants. The induction of systemic resistance was detected when the roots were colonized by the wild type or the *gac*S-complemented mutant but not by the *gac*S mutant (Han et al. 2006). Moreover, tobacco exposed to pure 2R, 3R-butanediol or to the volatiles emitted by *P. chlororaphis* O6 strain displayed increased leaf surface area, showing a positive effect on plant growth (Han et al. 2006). Mutations in the GacA/GacS regulatory system determined significant changes in the composition of volatile blend emitted by *P. chlororaphis* M71 strain. However, the synthesis of MT, that was the main component of the blend, was not affected by the mutations (Raio et al. 2020). Quorum sensing has also been suggested as regulatory system of bacterial VOC production in *P. aeruginosa* where the synthesis of 2-amino-acetophenone (2-AA) is under the control of the multiple virulence factor regulator (MvfR) that is an important QS regulatory system in acute infections (Kesarwani et al. 2011). 2-AA reduces the production of MvfR-regulated acute virulence factors, and attenuates acute virulence by negatively finetuning the MvfR regulon activity. Moreover, 2-AA adapts *P. aeruginosa* for chronic infections by promoting mutations in a key acute virulence gene (lasR) and by prolonging bacterial survival (Kesarwani et al. 2011).

### Mechanisms of action of microbial volatile compounds

mVOCs are involved in several direct and indirect interactions with plants such as pathogen inhibition (Aghdam et al. 2023; Dandurishvili et al. 2011; De Vrieze et al. 2015; Kai et al. 2007; Massawe et al. 2018; Raio et al. 2020; Raza et al. 2016), control of insects and nematodes (Deng et al. 2022; Popova et al. 2014; Song and Ryu 2013; Zhang et al. 2020), plant growth promotion (Cordovez et al. 2018; Park et al. 2015; Tahir et al. 2017) and induction of systemic resistance (Cho et al. 2008; Song and Ryu 2013). In the case of the fluorescent *P. tolaasii*, the causal agent of brown blotch disease on several edible mushrooms, the VOCs behaved as virulent factors by inhibiting the mycelium growth and were toxic toward basidiome tissues (Lo Cantore et al. 2015). Microbial volatiles may then interact with fungi, oomycetes and bacteria present in a given environments by modulating the growth, the production of secondary metabolites, the motility and biofilm formation, and virulence (Weisskopf et al. 2021). The modulation of different activities may concern the two microrganisms involved in the interaction through an interchange of bidirectional signals (Spraker et al. 2014). It is not clear how the target microbial cells receive the volatiles and so far, there is no evidence of the presence of receptors specific for these molecules (Weisskopf et al. 2021). Most probably, the physico-chemical properties of microbial volatiles allow passive diffusion through the cell wall or membrane (Weisskopf et al. 2021). It is important to underline that the biological activity of a single microbial volatile is not specific, in fact each molecule may interact with different micro- and marcro-organisms showing several functions (Table 1; Fig. 2). For example, cyanide is toxic to different micro- and macro- organisms (Table 1) and it is active by inhibiting cytochrome-c oxidase and then affecting the mitochondrial respiratory chain (Way 1984). DMDS is also able to inhibit different pathogenic bacteria and fungi (Aghdam et al. 2023; De Vrieze et al. 2015; Hernandez-Leon et al. 2015; Popova et al. 2014; Wang et al. 2020), to kill *D. melanogaster* (Popova et al. 2014) and may be also beneficial to plants by providing sulfur (Meldau et al. 2013).

Studies carried out to assay the effect of the single pure volatiles on different target microorganisms have shown interference at cellular level producing damages to fungal hyphae ultrastructures and conidia (Giorgio et al. 2015; Li et al. 2010), lethal effect on sporangia and lysis of zoospores in *Phytophthora* (De Vrieze et al. 2015), cell death and inhibition of biofilm formation in bacteria (Plyuta et al. 2016). The effect of microbial volatiles on plants is various and may be beneficial or deleterious depending on the molecule type and its concentration (Giorgio et al. 2015). Microbial volatiles may positively interact with plants through different mechanisms, one of which is related to their interference with the plant hormonal balance. In fact, in some cases, a change in the level of auxins, cytokinins, ethylene and abscissic acid has been observed (Bailly et al. 2014). Other studies have shown that the microbial volatiles may improve the ability of plants to absorb nutrients like iron and sulfur from the soil or to degrading sulfur volatile compounds for their own nutritional needs (Weisskopf et al. 2021). mVOCs are also involved in the induction of systemic resistance in plants, it is the case of 2,3 -butanediol produced by *P. chlororaphis* O6 that induced drought tolerance in *A. thaliana* by increasing stomatal closure and determining a consequent reduction of water loss (Cho et al. 2008).

### Identification of components of microbial volatile blends

Identification of mVOCs is carried out by using highly sensitive analytical techniques. Up to now, the methods described in literature vary greatly and there are still no standards or recommendations for the sampling and analysis of mVOCs (Wang et al. 2016). Gas chromatography coupled with mass spectrometry (GC-MS) is the most utilized analytical techniques to separate and identify mVOCs. VOC blend emitted by a bacterial culture is trapped on a cartridge filled with adsorbent material and then thermo-desorbed into a GC where the single molecules are separated before to be identified by a MS unit (Raio et al. 2020). In particular, molecule identification is achieved through the comparison of retention time and fragmentation pattern with available databases (Raio et al. 2020). GC-MS has largely been used to identify volatile compounds emitted by *Pseudomonas* species and the most recent studies often apply the SPME (solid phase microextraction) for sampling and GC-MS technique for analysis (Hernandez-Leon et al. 2015; Montes-Osuna et al. 2022; Raza et al. 2016; Wang et al. 2020). The proton transfer reaction mass spectrometry (PTR-MS) is a technique based on chemical ionization, where the reaction occurs between molecules of H_3_O^+^ and the VOCs present within a blend according to the proton affinity of VOCs. PTR-MS is a very sensitive technique allowing real-time detection of VOCs at concentrations down to ppt level (Wang et al. 2016). Both PTR-MS and GC-MS have been used to determine the variation of the VOC blend emitted by mutants of *P. chlororaphis* in comparison to the wild type strain (Raio et al. 2020). PTR-MS analysis allowed to determine the putative molecules emitted by the tested strains as percentage of the total blend without any pre-selection step. GC-MS analysis precisely identified the most representative molecules within the blend following selection of both the absorbent material used for sampling and the chromatographic column installed in the GC (Raio et al. 2020).

### Applications of microbial volatile compounds

There is a growing interest in the discovery and implementation of mVOCs as potential disease and pest management tools since only a little part of these compounds have been identified so far (Lemfack et al. 2018). Remarkable advances in understanding the relevance of mVOCs in microbe/microbes, microbe/insects and microbe/plants interactions have been done in recent years highlighting the important role of these molecules as biotechnological resources for a future sustainable and eco-friendly management of agricultural systems. In fact, the application of natural products in plant disease and pest management is strongly demanded as such products are better perceived by the public than synthetic chemicals in terms of safety and environmental sustainability (Qadri et al. 2020). Another possible application of mVOCs is to use them as biomarkers to recognize a target organism. For example, two mVOCs (isobutyl acetate and isoamyl alcohol) were found to be specific of the blend profile emitted by *Ceratocystis platani* a destructive fungal pathogen of plane trees. This finding could allow to exploit the mVOCs emitted by the pathogen to detect *C. platani* infected plants (Brilli et al. 2020) and it opens the possibility to use mVOCs fingerprinting as an innovative tool for the early diagnosis of plant diseases. The biotechnological exploitation of mVOCs is not restricted to agricultural applications. Recent studies exploring the possibility to use the microbial volatiles for identifying a single bacterial species within a mixed microbial culture, have been carried out on bacteria of clinical relevance (Lu et al. 2022). Specific volatiles emitted by *Escherichia coli*, *Pseudomonas aeruginosa* and *Staphylococcus aureus*, three species that normally infect wounds, were detected by gas chromatograph-ion migration spectroscopy (GC-IMS) a technique showing high sensitivity to identify and quantify specific VOCs. Specie-specific molecules were identified both from the pure cultures of single species and from the mixed culture, showing that the identification of specific mVOCs may represent a new diagnostic tool to detect the presence of a given species in infected wounds (Lu et al. 2022). However, in the real conditions the mixtures of microorganisms are complex, than the screening of specific volatiles should be not easy, also because the analytical techniques applied to this kind of studies produce a large amount of data to be elaborated.

## **The genus*Pseudomonas***

The genus *Pseudomonas* belongs to the class of Gamma *Proteobacteria* and is one of the most complex bacterial genera, since it currently includes the largest number of Gram-negative bacterial species (Gomila et al. 2015). The first studies on nucleic acid homology grouped the *Pseudomonas* members in five sharply defined rRNA groups one of which included *P. aeruginosa*, *P. fluorescens*, *P. putida*, *P. syringae* and the non-fluorescent species *P. stutzeri*, *P. alcaligenes*, *P. pseudoalcaligenes* and *P. mendocina*. The name *Pseudomonas* was then used to indicate this group of bacteria, named rRNA group I or *Pseudomonas* sensu stricto (Palleroni et al. 1973).

The taxonomy of *Pseudomonas* is in continuous evolution since new species are frequently identified and new methodologies allow to better discriminate the strains allocated in this genus. The analysis of the 16S rRNA gene sequence is a basic tool of the current bacterial classification system in fact, as a universal marker, it permits the ascription of a strain to the genus and allows comparisons between very divergent bacteria (Lalucat et al. 2020). However, the resolution of 16S rRNA gene sequence at the intrageneric level is low (Gomila et al. 2015; Mulet et al. 2010; Yamamoto et al. 2000). The utility of other house-keeping core genes for phylogenetic studies has been demonstrated and has been largely used for *Pseudomonas*. These genes evolve more slowly than typical protein-coding genes but more rapidly than rRNA genes (Mulet et al. 2010). The multilocus sequence analysis (MLSA) based on the four housekeeping genes 16S rRNA, *gyr*B, *rpo*B and *rpo*D sequencing performed on 107 *Pseudomonas* type strains, allowed the discrimination of two intrageneric groups (IG), one of which named IG *P. fluorescens*, including nine subgroups, and the other was named IG *P. aeruginosa* (Mulet et al. 2010). MLSA of the four housekeeping genes was later performed both on the genomes available in public databases and the type strains belonging to the *P. fluorescens* group using *P. aeruginosa* as outgroup species (Garrido-Sanz et al. 2016). Based on this analysis, *P. fluorescens* group was divided in nine subgroups (*P. protegens*, *P. chlororaphis*, *P. corrugata*, *P. koreensis*, *P. jessenii*, *P. mandelii*, *P. fragi*, *P. gessardii* and *P. fluorescens*) and was named “*P. fluorescens* complex”. This is one of the most diverse groups within the *Pseudomonas* genus, comprising both validly named species and many unclassified isolates (Garrido-Sanz et al. 2017). Many strains of *P. fluorescens* complex have been isolated from plant-related environments and several species can be considered beneficial by directly and indirectly inhibiting pathogen activities and promoting plant health (Arrebola et al. 2019). The analysis of each group-specific genome and the search for key features revealed congruence between the phylogenomic determination of these groups and their ecophysiology. *P. corrugata*, *P. protegens* and *P. chlororaphis* groups include the most effective strains for biocontrol while *P. jessenii* group is more suitable for bioremediation/rhizoremediation applications (Garrido-Sanz et al. 2016). A more recent phylogenetic study of the genus *Pseudomonas*, based on analysis of gene sequence or whole genome comparison using different algorithms, showed that several named species are synonymous, many strains assigned to known species have to be proposed as new genomic species while the main phylogenetic groups defined by MLSA are concordant with the clustering obtained by whole genome analysis (Lalucat et al. 2020). The Whole Genome Sequencing (WGS) technologies has increased the number of publicly available genomes and, therefore, genome-to genome comparisons, with the Average Nucleotide Identity and digital DNA–DNA hybridization have become standards for species definition (Girard et al. 2021). Consequently, new *Pseudomonas* species are frequently reported and the number of species within the genus regularly increases. Moreover, whole genome sequencing allows to update the *Pseudomonas* evolutionary and taxonomic relationships (Girard et al. 2021).

This review will consider the bacteria belonging to the *P. fluorescens* complex as described by Garrido-Sanz et al. (2016) since many species beneficial to plants acting through different mechanisms are included in this group. In addition, the chemical composition of the blend of volatiles emitted by different strains is known, the effect of single molecules on different organisms has been investigated and then a remarkable literature production is available on this topic.

## **Plant pathogen control by*Pseudomonas*volatile compounds**

### Fungi control by *Pseudomonas* volatile compounds

Many studies regard the control of several phytopathogenic fungi by mVOCs emitted by different species of the *P. fluorescens* complex and the identification of specific molecules involved in the interaction with the target pathogens (Athukorala et al. 2010; Cordero et al. 2014; Giorgio et al. 2015; Hernandez-Leon et al. 2015; Kai et al. 2007; Ni et al. 2022; Popova et al. 2014; Raio et al. 2020; Strano et al. 2017; Wang et al. 2021; Zhang et al. 2019; Zhong et al. 2021). *Rhizoctonia solani* growth was inhibited by 1-undecene emitted by *P. fluorescens* and *P. trivialis* (Kai et al. 2007) and by 2-nonanone and 2-undecanone (Popova et al. 2014) emitted by *P. chlororaphis* 449 strain. mVOCs emitted by different *P. fluorescens* strains were effective against *Botrytis cinerea in vitro* as pure compounds (DMDS, DMTS, geranyl formate, acetic acid, butyric acid, 2-methylbutyric acid, isobutyric acid, isovaleric acid) by inhibiting the mycelial growth (Zhong et al. 2021). mVOCs applied in vivo prevented gray mold development on grapes by fumigation (Zhong et al. 2021) and reduced stem disease symptoms and root browning on *Medicago truncatula* plants (Hernandez-Leon et al. 2015). In addition, HCN, synthesized by *P. corrugata* CFBP5454 and *P. mediterranea* CFBP 5457 strains was instead responsible of the inhibition of *B. cynerea* conidium germination (Strano et al. 2017). The VOCs from *P. fluorescens* ZX also inhibited mycelial growth and conidial germination of *Penicillim italicum* and effectively controlled the blue mould decay in vivo: organic acids and sulfur compounds were the active components of VOCs, with DMDS and DMTS exhibiting the highest antifungal activity (Wang et al. 2021). Several *P. chlororaphis* strains have been studied for their ability to produce volatile compounds effective against phytopatogenic fungi. Three alcohol (3-methyl-1-butanol, phenylethylalcohol, 2-methyl-1-butanol) resulted the most effective in inhibiting the mycelial growth of *C. fimbriata in vitro* (Zhang et al. 2019). Inhibition of mycelium growth and sclerotia germination of *S. sclerotiorum in vitro* was determined by acetic acid and 2-nonanone produced by *P. brassicacearum* (Giorgio et al. 2015). In fact, these pure VOCs showed the lowest value of minimal inhibitory quantity necessary to reduce mycelium growth (4,19 and 4,92 mg respectively) and sclerotia germination (9,44 and 16, 4 mg). The strain of *P. chlororaphis* PA23 produced the volatiles nonanal, benzothiazole and 2-ethyl-1-hexanol that were involved, in combination with a phenazine, in the control of *Sclerotinia sclerotiorum* on sunflower in vivo (Athukorala et al. 2010). Similarly, the biocontrol of *Seiridium cardinale* by *P. chlororaphis* M71 strain was due to the action of the antibiotic phenazine-1-carboxylic acid and to the emission of volatile compounds (Raio et al. 2017, 2020). Noteworthy, the blend of volatile compounds emitted by *P. chlororaphis* M71 strain inhibited in vitro only *S. cardinale* (Fig. 2) out of the six fungal phytopathogens tested. MT was the main volatile compound detected in the blend of M71 strain but the effectiveness of the pure molecule in controlling *S. cardinale* was not assayed (Raio et al. 2020). *P. aurantiaca* ST-TJ4 strain inhibited the radial growth, conidia germination and microsclerotia formation of *Verticillium dahliae*, strongly reduced the symptoms on the cotton seedling roots and the expression of genes related to melanin synthesis in *V. dahliae* (Ni et al. 2022). The volatiles 2-undecanone and 1-nonanol, assayed as pure compounds, effectively inhibited the *V. dahliae* radial growth (Ni et al. 2022). VOCs of *P. protegens* CHAO strain negatively affected the growth of the fungus *Heterobasidion abietinum*. DMTS, 2-ethylhexanol and ammonia were involved in this interaction determining severe cellular and subcellular alterations of hyphae (Prigigallo et al. 2021).

A particular group of volatile compounds were identified from the blend of *P. veronii* R02 strain, in fact for the first time, long chain aliphatic nitriles were found and among these, methyltridec-3-enenitrile showed a strong activity against *Mucor hiemalis*. This represents the first report regarding the retrievement of this class of molecules in nature (Montes Vidal et al. 2017) and evidences that further new volatiles emitted by little investigated *Pseudomonas* species may be identified.

### Oomycete control by *Pseudomonas* volatile compounds

Few studies have been conducted so far on the efficacy of mVOCs in controlling oomycetes. An anti-oomycete activity related to volatile emission, was reported for fluorescent pseudomonads isolated from potato (De Vrieze et al. 2015; Hunziker et al. 2015). 1-Undecene was the main compound detected for *P. chlororaphis* R47 and *P. fluorescens* R76. This compound determined a dose-dependent inhibition of mycelium growth associated to a reduction of sporangia number and zoospore release in *Phytophthora infestans* (Hunziker et al. 2015). De Vrieze et al. (2015) confirmed this result regarding 1-undecene, however the doses required to achieve significant *P. infestans* growth inhibition were considered too high (> 1 mg). Other VOCs included in the blend profile of the two *Pseudomonas* strains, such as nitropentane, isovaleric acid, undecanal, DMTS, MMTS, propiophenone and phenylpropanedione, determined a total inhibition of *P. infestans* growth and had a heavy negative impact on sporangia production and zoospore release at the dose below 1 mg (De Vrieze et al. 2015). The emission of 1-undecene and sulfur compounds was also demonstrated in vivo by inoculating potato leaves with the VOC producing *Pseudomonas* strains (Gfeller et al. 2022). The nutrients present on potato leaves were sufficient to allow the growth of the bacterial strains and the production of volatile compounds able to inhibit *P. infestans* (Gfeller et al. 2022). These findings strongly support the possibility to successfully transfer in vivo the results of in vitro control of phytopathogens by mVOCs. A study regarding the control of *Phytophthora cinnamomi* showed that *P. aurantiaca* ST-TJ4 interacted with the pathogen by multiple mechanisms of biocontrol involving against phenazines, ammonia and 2-undecanone (Zhang et al. 2022).

### Bacteria control by *Pseudomonas* volatile compounds

**Fig. 3:**
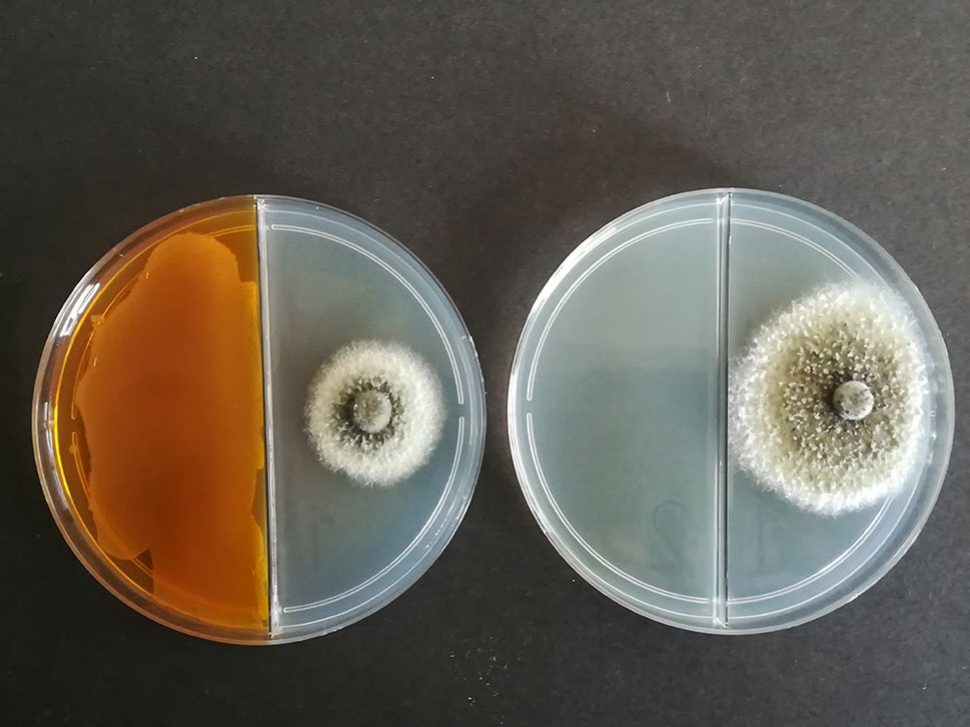
Effect of mVOC blend emitted by *Pseudomonas chlororaphis* M71 strain on *Seiridium cardinale*. mVOCs emitted by *Pseudomonas chlororaphis* M71 strain significantly reduced the radial growth of *Seiridium cardinale* colony (**a**) in comparison to the control (**b**)

Many studies carried out on the effect of mVOCs produced by *Pseudomonas* on phtopathogenic bacterial species have regarded *Agrobacterium tumefaciens* and have evidenced bacteriostatic or bactericidal activities, ability in reducing tumor development and interference with motility and biofilm formation. Strains of *P. fluorescens* significantly reduced the development of tumors on tomato stems artificially inoculated with *A. tumefaciens* and the application of pure molecules that composed the VOC blend emitted by the strains showed that DMDS was able to suppress the bacterium growth (Dandurishivili et al. 2011). DMDS was also responsible of a bacteriostatic effect on *A. tumefaciens* grown in dual culture assay with *P. chlororaphis* strain 449 (Popova et al. 2014). The individual VOCs produced by two *Pseudomonas* strains, (2-nonanone, 2-heptanone, 2-undecanone) and DMDS produced by *Serratia* strains, killed *A. tumefaciens* cells in mature biofilms and suppressed the biofilm formation, showing that the lack of bacterial growth was the main factor responsible of the biofilm formation inhibition (Plyuta et al. 2016). The effect on bacterial biofilm increased with the concentration of the pure VOCs tested. For example, increasing the concentration of 2-nonanone from 5 µmol to 35 µmol led to 10-fold decrease in the number of living cells in mature biofilms of *Agrobacterium* strains (Plyuta et al. 2016). A marked inhibition of motility and biofilm formation ability in *A. tumefaciens* was also induced by two strains of *P. kilonensis* isolated as endophytes of grapevine plants (Asghari et al. 2019; Etminani et al. 2022). These strains were able to produce long-chain alkenes (dodecane, tetradecane, hexadecane) and aromatic compounds that also determined a decreasing effect on gall weight and various alterations on *A. tumefaciens* cell morphology (Etminani et al. 2022). These data suggest an additional potential of some ketones and DMDS as protectors of plants against *A. tumefaciens* strains. In fact, the ability of phytopathogenic bacteria to form biofilms on plants, including *Agrobacterium* species, is one of the virulence factors involved in bacterial infection process because in a biofilm state, bacteria are more resistant to plant defensive mechanisms and are proficient in plant colonization. The VOCs previously listed also suppressed the swimming motility of agrobacteria (Sidorova et al. 2022). It is known that some volatile organic compounds (1-butanol, 2-butanone, acetoin, ethanol, hexadecane, glyoxylic acid) slightly influenced, positively or negatively, biofilm formation in *E. coli*, *Pseudomonas aeruginosa*, *Staphylococcus aureus* and *Bacillus subtilis*. Some of them increased or decreased the motility of these bacteria, a trait important for the formation of biofilms, plant colonization and entering plant tissues during infection (Audrain et al. 2015) (see Fig. 3).

**Fig. 4:**
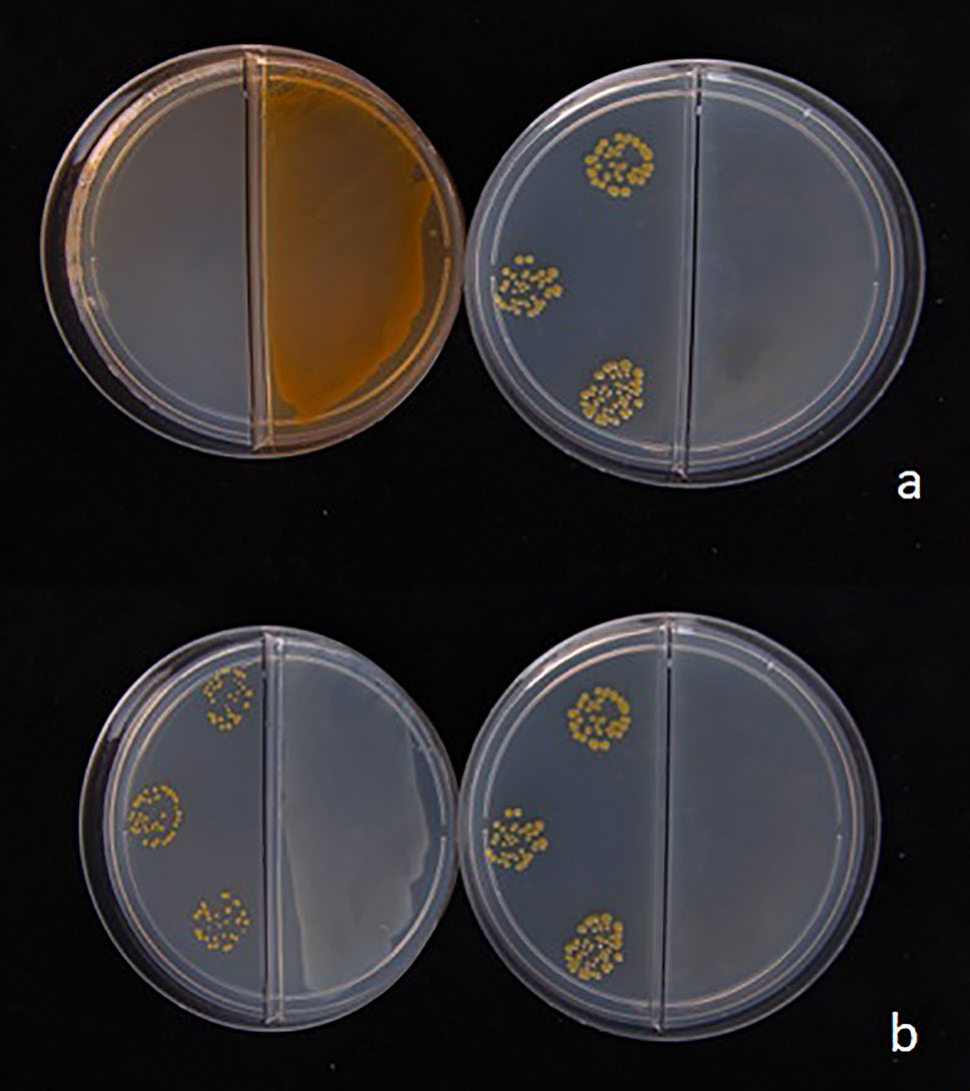
Effect of mVOCs emitted by *Pseudomonas chlororaphis* M71 strain on *Clavibacter michiganensis* subsp. *michiganensis*. **a** M71 wild type strain (left plate) completely inhibited the *C. michiganensis* subsp. *michiganensis* colony growth in comparison to control (right plate); **b** M71 gacA mutant did not inhibited the *C. michiganensis* subsp. *michiganensis* colony growth (left plate), but determined only a slight reduction of the colony size in comparison to the control (right plate)

VOCs of *P. chlororaphis* M71 strain completely inhibited the development of colonies of *Clavibacter michiganensis* subsp. *michiganensis in vitro* (Fig. 4) but were ineffective against *Rhodococcus fascians* (Raio et al. 2020). The M71 *gac*A mutant did not inhibited the bacterial pathogen growth, but determined only a reduction of colony size. Since the synthesis of HCN was significantly reduced in this mutant, it was hypothesized that this compound was involved in the inhibition of *C. michiganensis* subsp. *michiganensis* growth by M71 wild type strain (Raio et al. 2020). Volatiles may also interact with virulence factors of phtypathogenic bacteria as described for *Ralstonia solanacearum* where the expression of 68 cellular proteins was changed significantly after exposure to the VOCs of *P. fluorescens* WR-1. Among these, proteins involved in the antioxidant activity were downregulated while three ABC transporter system proteins were upregulated (Raza et al. 2016). Volatiles emitted by *P. brassicacearum* EnPb strain, isolated as endophyte from a potato tuber, significantly reduced the development of *Pectobacterium carotovorum* subsp. *carotovorum* colonies in vitro and the damage determined by the pathogen on potato tubers under storage conditions (Aghdam et al. 2023). The antagonist was able to produce 2, 4-diacetylphloroglucinol, lytic enzymes, DMDS and DMTS then it is plausible that a combination of several mechanisms were involved in the biocontrol activity of *P. brassicacearum* EnPb strain against *P. carotovorum* subsp. *carotovorum* (Aghdam et al. 2023) as described previously for some fungal pathogens.

## Nematode and insect control by *Pseudomonas* volatile compounds

Volatile compounds emitted by some fluorescent *Pseudomonas* species have been also evaluated for their nematicidal and insecticidal activity. 1-Undecene inhibited the development of *Caenorhabditis elegans* and reduced its viability in a time and dose/time-dependent way, in fact a 25 µmol concentration was the minimum dose needed to detect a partial inhibition of the nematode vitality, while 100 µmol killed all nematodes in few days (Popova et al. 2014). HCN was instead the primary compound involved in *C. elegans* intoxication caused by *P. chlororaphis* PA23 strain, moreover a synergic effect of HCN and the non-volatile antibiotic pyrrolnitrin produced by PA23 strain was observed against the nematode (Nandi et al. 2015). Similarly, *P. brassicacearum* DF41 strain drastically reduced the viability of *C. elegans* by HCN emission (Nandi et al. 2016). However, DF41 strain was also able to form biofilm on the nematode head which blocked feeding and caused starvation. When co-cultured with *C. elegans*, DF41 exhibits altered gene expression and metabolite production, indicating that it can sense the presence of the bacterium predator and adjust its physiology accordingly (Nandi et al. 2016).

1-Undecene, DMDS, 2-nonanone and 2-heptanone were the main volatiles produced by *P. chlororaphis* 449 and as pure compounds they had strong killing effect on *Drosophila melanogaster* at 5–10 µmol (Popova et al. 2014). HCN is one of the components of the anti-insect arsenal common to *P. chlororaphis* and *P. protegens* groups (Flury et al. 2017). In fact, HCN was essential to full virulence of *P. chlororaphis* PCL1391 and *P. protegens* CHA0 strains directly injected into the hemolymph of *Galleria mellonella* and *Plutella xylostella* larvae, while cyclic lipopeptides played a major role during the oral infection of larvae (Flury et al. 2017). In relation with insects, mVOCs may play also different ecological roles by signalling habitat suitability or exposure to enthomopathogens, inducing insect aggregation or eliciting mating and ovideposition, acting as sexual pherormones (Davis et al. 2013).

## Plant growth promotion and induction of defense mechanisms by *Pseudomonas* volatile compounds

mVOCs may modulate plant physiological and hormonal pathways resulting in an increased biomass and yield production (Sharifi and Ryu 2018). Changes in plant morphological traits such as root volume, leaf number and size, flower number, fruit and seed production are the common parameters considered to evaluate the plant growth promotion ability of microrganisms. mVOCs are also able to improve plant health acting as elicitors of plant immunity through the salicylic acid (SA) and jasmonic acid pathways (Cho et al. 2008; Cortes-Barco et al. 2010; Lee et al. 2012; Ryu et al. 2004; van de Mortel et al. 2012). Although many molecular mechanisms underlying mVOCs perception by plant still remain unclear, numerous studies have demonstrated that these molecules may determine a potent priming of the plant basal immune system, termed induced systemic resistance (ISR). Many ISR-triggering microorganisms have been investigated for their plant growth-promoting and stress-relieving properties (Bailly and Weisskopf 2017; Blom et al. 2011; Cordovez et al. 2018; Poveda 2021). Volatile compounds produced by bacteria belonging to *Pseudomonas* and *Bacillus* genera are the most investigated and *A. thaliana* is the plant system chosen to carry out most researches on mVOCs effects and then has been proposed as model to study the interactions with plants (Li et al. 2019). Many volatile compounds have been found as shared components of the blends emitted by different microbes while some molecules have been found associated to unique species (Jishma et al. 2017; Montes Vidal et al. 2017). The volatiles acetoin and 2R,3R-butanediol were shown to promote growth in *A.thaliana* where a marked enhancement of leaf surface area was observed (Ryu et al. 2003). Later, it was shown that exposition of *A.thaliana* plants to mVOCs upregulated gene expression for auxin synthesis and auxin accumulation that decreased in leaves and increased in roots. Moreover, leaf cell expansion due to cell wall loosening was also induced (Zhang et al. 2007). 2R,3R-butanediol is the main volatile produced by *P. chlororaphis* O6 strain involved in induction of systemic response in tobacco against the pathogenic bacterium *E. carotovora* (Han et al. 2006). The mechanism of the response activation was unknown and was effective against *E. carotovora* but not against *Pseudomonas syringae* pv. *tabaci* (Han et al. 2006). MT and DMDS effect on broccoli and lettuce seed germination and seedling growth varied according to the dose applied. DMDS at doses lower than 1 µg stimulated the seedling growth, while inhibition was determined by a dose of 2,5 µg. A stimulation of seedling growth was also oserved for 1-undecene at a concentration higher than 10 µg (Lo Cantore et al. 2015). This hydrocarbon can be assimilated or transformed by microorganisms but the mechanisms related to its effect on plant growth was not clarified (Lo Cantore et al. 2015).

Colonization of *A. thaliana* roots by the rhizobacterium *P. fluorescens* SS101 leaded to an enhanced level of resistance against various bacterial pathogens including *Pseudomonas syringae* pv. *tomato* and the lepidopteran insect herbivore *Spodoptera exigua*. Metabolomics and transcriptomics data showed that this specific rhizobacterial strain induces extensive metabolic and transcriptional changes in roots and leaves of *A. thaliana* and that glucosinolates and camalexin play important roles in the resistance response of *A. thaliana* induced by SS101 (van de Mortel et al. 2012). The same strain was also able to enhance the growth of tobacco plants through the emission of a complex blend of volatiles. The application of pure 13-tetradecadien-1-ol, 2-butanone and 2-methyl-n-1-tridecene to tobacco plants evidenced the major involvement of 13-tetradecadien-1-ol in growth promotion (Park et al. 2015). Four strains of *P. fluorescens* showed both a marked biocontrol activity against *B. cinerea* and growth promotion induction in *Medicago truncatula*. The main VOCs emitted by all four strains were the sulfur-containing volatiles MT, DMS, DMDS and DMTS (Hernandez-Leon et al. 2015) and DMDS was the most abundant VOC detected. This molecule is known for high antifungal activity and also as elicitor factor of systemic resistance in plants (Huang et al. 2012). One of the four strains produced DMHDA which showed antifungal properties and also increased biomass, chlorophyll and iron content in *M. truncatula* plants (Hernandez-Leon et al. 2015). The VOC blend of the fluorescent *Pseudomonas* PICF7 strain was instead investigated in consideration of its ability to control the olive pathogens *Verticillium dahliae* and *Verticillium longisporum* (Montes-Osuna et al. 2022). Interestingly, the volatiles emitted by PICF7 strain were different when the antagonist was grown in presence of the fungal pathogen (Montes-Osuna et al. 2022), showing that to understand the effective role of mVOCs in plant protection, investigations have to be performed considering the complex microbe-plant interactions. In fact, various VOCs may exert different actions on plants, depending on many factors including the plant species, the conditions of their growth and the amount of the tested volatile compound (Plyuta et al. 2021). Few examples regard the induction of systemic tolerance to abiotic factors due to *Pseudomonas* mVOCs. 2R,3R-butanediol was a major determinant in reducing water loss in *P. chlororaphis* O6-colonized plants acting through stomatal closure regulation (Cho et al. 2008). Later, it was demonstrated that the production of nitric oxide and hydrogen peroxide stimulated by 2,3-butanediol, induced systemic tolerance to drought in *A. thaliana* through an SA-dependent response (Cho et al. 2013). The mVOCs emitted by *P. simiae* strain AU enhanced soybean seedling growth and induced systemic tolerance to high sodium conditions (Vaishnav et al. 2015). A reduction of Na^+^ ions in root and shoot and an increase of proline and chlorophyll content was induced by the exposition of plants to the volatiles emitted by the AU strain. These changes allowed the plants to maintain the osmotic balance and increased the photosynthesis activity under salt stress (Vaishnav et al. 2015). Some studies evidenced a negative effect of mVOCs on plant growth. Drastic inhibition was observed growing *A. thaliana *plants in presence of the volatiles emitted by *P. fluorescens* and *P. trivialis* strains (Vespermann et al. 2007). Volatiles emitted by *P. chlororaphis* 449 strain inhibited *A. thaliana* growth in vitro. DMDS and the ketones 2-nonanone and 2-undecanone had a plant-killing effect. HCN was instead not responsible of the *A. thaliana* growth inhibition as the *rpo*S mutant, unable to synthesize HCN, conserved the plant killing effect (Plyuta et al. 2021).

## Conclusions and outlook

*P. fluorescens* complex is a high diverse group of bacteria within the *Pseudomonas* genus, including several beneficial species living in plant-related environments. During the last decades a great research effort has been made to investigate the role of microbial volatile compounds in plant protection both as antimicrobials and as molecules triggering systemic resistance against biotic and abiotic factors. These studies have shown the great potential of these molecules to be exploited as plant protectants. The mVOC blends emitted by strains belonging to different species of fluorescent *Pseudomonas* have been tested for their ability to control plant pathogens and pests and also for promoting growth or inducing defense responses in plants. mVOCs of *P. fluorescens* and *P. chlororaphis* are the most investigated since these bacteria are highly effective biocontrol agents acting through various mechanisms (antibiosis, siderophore, lytic enzymes) and able to actively colonize the plant rhizosphere and phyllosphere. Most of the research performed so far has concerned the determination of the antimicrobial activity of the total blends or of the single pure compounds against phytopathogenic fungi and bacteria, while a low number of researches has been done on the induction of defense responses and mechanisms involved in these interactions. To date, most studies have been focused on morphological and phenotypical changes induced on plants by mVOCs, however, the mechanisms involved are still poorly explored. Further studies are needed to understand how the volatile compounds penetrate the plant tissues and are perceived by plant cells, to evidence the putative transport through the plants and the changes that happen at molecular level (Poveda 2021). A great diversity in the composition of the mVOC blends produced by different species/strains of fluorescent *Pseudomonas* has been described so far. Indeed, molecules belonging to different chemical classes have been identified, among which alkenes, sulfur compounds, alcohols, ketones, organic acids and inorganic compounds are the most frequent (Fig. 1). The wide variability observed is mainly related to the microbial metabolic capacity and to the availability and composition of the substrate (Piechulla et al. 2020). Substrate availability in the laboratory tests and in nature is considerably different, therefore mVOC profiles of bacteria grown under laboratory conditions are most likely different from those of the same bacteria transferred to an agricultural or natural environment (Piechulla et al. 2020). Most results were obtained using the dual culture method in split petri dishes where the target microorganism is treated with a complex mixture of volatiles of the putative emitter or a pure molecule. This method strongly simplifies the interaction since the numerous factors present in the natural environment (nutrient availability, bacterial concentration, interaction with other micro- and macro-organisms, temperature, etc.) are not taken into consideration and therefore it is useful to perform a rapid screen only. However, it is clear that under natural conditions, the behavior of the emitter and receiver organisms will be different and it is realistic to assume that the result of the interaction will be not the same as observed under controlled conditions. Moreover, the experimental approach used so far is not standardized, indeed the published results have been obtained performing the tests under several different lab conditions and sometimes contrasting results have also been reported. One of the future challenges will be to set up standardized procedures that will simulate as much as possible the natural environment in order to unravel the biological role of mVOCs.

The wide research interest around the mVOCs is related to the growing demand of new bioactive compounds for agriculture applications (but not exclusively), showing low environmental impact and safe for human and animals. During the last years several chemicals employed in agriculture as pesticides, herbicides etc., have been banned given their toxicity and accumulation in the environment (water, soil and air pollution), therefore the search for new molecules and new formulations environment friendly are strongly needed. Based on literature available, at the present about 2000 mVOCs have been identified from nearly 1000 fungal and bacterial species. In consideration that 10^18^ microbial species are expected to exist on earth, many novel mVOCs will be identified in the future (Lemfack et al. 2018). mVOCs may therefore represent an enormous resource of new molecules of natural origin, to be exploited for setting up safe crop protection strategies.

A crucial aspect regarding the practical application of mVOCs is the transfer of the results obtained under laboratory conditions, to the agri-food chain. Actually, the best way for a successful application of mVOCs is the control of post-harvest diseases since the treatment is performed in a confined environment and under controlled parameters and the direct contact of the molecules with the crops is not required (Li et al. 2010; Wang et al. 2020). The use of volatiles for open field treatments presents several drawbacks since the efficacy of the single molecules or artificial blends can be affected by the multiple biotic and abiotic factors present in the agricultural/natural environment that may even invalidate the beneficial effects observed during the experimental step. However, the effectiveness of some bacterial mVOCs as inducer of ISR has been evaluated under field conditions. It is the case of 3-pentanol, which applied on the roots of pepper plants induced the resistance against *Xanthomonas axonopodis* pv. *vesicatoria* the responsible of bacterial spot disease (Choi et al. 2014); moreover, systemic resistance to cucumber and tobacco mosaic virus was observed on pepper plants grown in soil drenched with 2, 3-butanediol, both under greenhouse and field conditions (Kong et al. 2018). Some commercial products DMDS-based, are now available as soil fumigants with a wide spectrum of activity (nematicide, herbicide and fungicide). (https://www.arkema.com/global/en/products/product-finder/product-range/thiochemicals/dmds-for-agricultural-soil-fumig/).

The low solubility in water and high volatility strongly affect the over-time efficacy of mVOCs. Engineered strategies have been applied for obtaining microbial strains able to synthesize high concentrations of specific volatiles. For example, through a modification of the biosynthetic pathway, it was obtained an over production of the terpenoid β-ionone in the yeast *Yarrowia lipolytica* (Lu et al. 2020). However, to the present no *Pseudomonas* strains genetically modified for volatile compound overproduction have been constructed, but this may represent a future research challenge. Innovative technologies also may help to overcome low solubility in water and high volatility features and allow a controlled release of the active molecules. For example, cocrystallization was used to tune the physical and delivery properties of essential oils in order to extend their range of applicability in agriculture (Mazzeo et al. 2019). A wide commercial application of mVOCs is desirable and it is predictable that the current huge research efforts focused on this topic will bring to the development of new eco-friendly strategies for plant protection in the next future.

## Data Availability

The datasets generated during and/or analysed during the current study are available from the corresponding author on reasonable request.

## Abbreviations

DMS: Dimethyl sulphide
DMDS: Dimethyl disulfide
DMHDA: Dimethylhexadecilamine
DMTS: Dimethyl trisulfide
HCN: Hydrogen cyanide
MMTS: S-methyl methanethiosulfonate
MT: Methanethiol
VOCs: Volatile organic compounds
